# Recombinant Human Secretory IgA Induces *Salmonella* Typhimurium Agglutination and Limits Bacterial Invasion into Gut-Associated Lymphoid Tissues

**DOI:** 10.1021/acsinfecdis.0c00842

**Published:** 2021-03-17

**Authors:** Angelene F. Richards, Danielle E. Baranova, Matteo S. Pizzuto, Stefano Jaconi, Graham G. Willsey, Fernando J. Torres-Velez, Jennifer E. Doering, Fabio Benigni, Davide Corti, Nicholas J. Mantis

**Affiliations:** † Department of Biomedical Sciences, University at Albany School of Public Health, Albany, New York 12208, United States; ‡ Division of Infectious Diseases, Wadsworth Center, New York State Department of Health, Albany, New York 12208, United States; ∥ Humabs BioMed SA a Subsidiary of Vir Biotechnology Inc., 6500 Bellinzona, Switzerland

**Keywords:** immunity, antibody, secretory immunoglobulin A, *Salmonella enterica*, Peyer’s patch

## Abstract

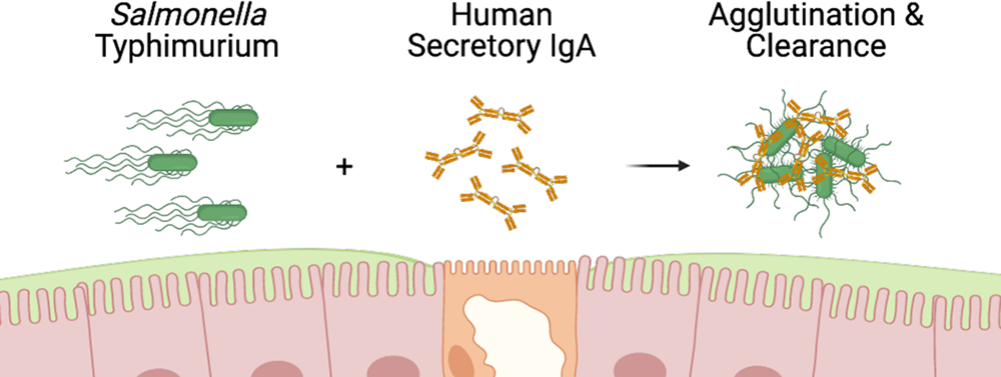

As the predominant antibody type in mucosal secretions, human colostrum, and breast milk, secretory IgA (SIgA) plays a central role in safeguarding the intestinal epithelium of newborns from invasive enteric pathogens like the Gram-negative bacterium *Salmonella enterica* serovar Typhimurium (STm). SIgA is a complex molecule, consisting of an assemblage of two or more IgA monomers, joining (J)-chain, and secretory component (SC), whose exact functions in neutralizing pathogens are only beginning to be elucidated. In this study, we produced and characterized a recombinant human SIgA variant of Sal4, a well-characterized monoclonal antibody (mAb) specific for the O5-antigen of STm lipopolysaccharide (LPS). We demonstrate by flow cytometry, light microscopy, and fluorescence microscopy that Sal4 SIgA promotes the formation of large, densely packed bacterial aggregates *in vitro*. In a mouse model, passive oral administration of Sal4 SIgA was sufficient to entrap STm within the intestinal lumen and reduce bacterial invasion into gut-associated lymphoid tissues by several orders of magnitude. Bacterial aggregates induced by Sal4 SIgA treatment in the intestinal lumen were recalcitrant to immunohistochemical staining, suggesting the bacteria were encased in a protective capsule. Indeed, a crystal violet staining assay demonstrated that STm secretes an extracellular matrix enriched in cellulose following even short exposures to Sal4 SIgA. Collectively, these results demonstrate that recombinant human SIgA recapitulates key biological activities associated with mucosal immunity and raises the prospect of oral passive immunization to combat enteric diseases.

Globally, diarrheal diseases remain a leading cause of morbidity and mortality among children.^1^ The Global Burden of Diseases (GBD) investigative group reported in 2019, for example, that gastrointestinal infections resulting in prolonged intestinal inflammation and malnutrition were the third leading cause of disability-adjusted-life years (DALY) lost by children under the age of ten.^2^ The majority of these cases are concentrated within regions of high socioeconomic disparity, such as Sub-Saharan Africa and Southeast Asia.^3^ A number of enteric pathogens have been attributed to high diarrheal incidence in these areas, including enterotoxigenic *Escherichia coli* (ETEC), *Shigella* sp., *Campylobacter jejuni*, and *Vibrio cholerae*.^4^*Salmonella enterica* is also on the list of pathogen-specific sources of severe diarrhea, accounting for 95.1 million cases worldwide in 2017.^5^ While nontyphoidal *Salmonella* serovars typically cause self-limiting gastroenteritis, there is an emergence of invasive nontyphoidal strains (iNTS) that cause severe systemic infection.^6^ iNTS are associated with antibiotic resistance and increased mortality rates, often disproportionately impacting vulnerable populations of HIV-infected adults and young children.^6−8^

Secretory IgA (SIgA) is the predominant immunoglobulin on mucosal surfaces and serves as a formidable barrier against bacterial and viral pathogens. SIgA is also the primary antibody found in human colostrum and breast milk.^9,10^ At its core, SIgA consists of two IgA monomers (mIgA) covalently attached at their C-termini by joining (J)-chain (15 kDa).^11−13^ Humans have two IgA isotypes, IgA1 and IgA2, that differ structurally in their hinge regions and degrees of O-glycosylation.^14^ Dimeric (dIgA) and some higher molecular weight polymers (pIgA) are produced by plasma cells in the intestinal lamina propria. Dimeric IgA is selectively transported across the intestinal epithelium in a basolateral-to-apical direction by the polymeric immunoglobulin (pIgR) receptor.^15^ Following transcytosis, the ectodomain of pIgR is proteolytically cleaved and remains associated with the Fc regions of dIgA, generating a complex known as SIgA.^12,16^ The cleaved ectodomain of pIgR is referred to as secretory component (SC).^17^ pIgR is also expressed in mammary epithelial tissues and is responsible for the delivery of IgA into colostrum and breast milk.

Once in mucosal secretions and breast milk, SIgA is proposed to protect the intestinal epithelium through a process known as “immune exclusion” in which SIgA promotes antigen and pathogen cross-linking, entrapment in the intestinal lumen, and eventual clearance from the gastrointestinal tract through peristalsis.^15,18,19^ By restricting access to the intestinal epithelium, SIgA effectively prevents pathogens like *E. coli*, *C. jejuni*, and iNTS from colonizing and invading the gut mucosa. SIgA is uniquely suited to perform this function, as the molecule is surrounded by a “glycan shield”.^20^ SC alone has seven N-linked glycosylation sites.^21,22^ The substantial glycosylation renders SIgA relatively stable in the acidic and proteolytic conditions of the gut, compared to other antibody isotypes like IgG.^23−26^ The extensive carbohydrate side chains also anchor SIgA in the intestinal mucus on epithelial surfaces.^27,28^

These unique attributes have garnered interest in SIgA as an alternative or supplement to antibiotic-based therapeutics for enteric diseases. Recent advancements in the generation of recombinant antibodies via multivector mammalian expression systems have enabled the production of SIgA monoclonal antibodies (mAbs).^29−31^While there are examples in which passively administered mouse IgA or SIgA has been shown to afford immunity against experimental shigellosis and cholera, the use of human SIgA is only beginning to be explored.^32−35^ For example, we recently showed in a mouse model of invasive *Salmonella* infection that Sal4 IgA, an anti-lipopolysaccharide mouse mAb, was sufficient at reducing the invasion of *Salmonella enterica* serovar Typhimurium (STm) into Peyer’s patch tissues.^36^ Sal4 SIgA was superior to Sal4 IgG.^36^ Our results demonstrated that, while both Sal4 isotypes were functional *in vitro*, only Sal4 mouse IgA was able to prevent STm from entry and dissemination in the mouse gut.

In this report, we sought to examine whether a human SIgA version of Sal4 is also effective at limiting STm infection in the mouse model. We were prompted by recent studies from other groups showing that human SIgA mAbs specific for a bacterial adhesin and flagellar protein subunits prevent intestinal colonization from ETEC and *C. jejuni*, respectively.^30,37^ We report that human recombinant Sal4 SIgA is a potent inducer of STm agglutination *in vitro* and *in vivo* and that this activity likely contributes to bacterial entrapment in intestinal lumen and limits invasion into gut-associated lymphoid tissues.

## Results

### Recombinant Human Sal4 SIgA Induces STm Agglutination

**Figure 1:**
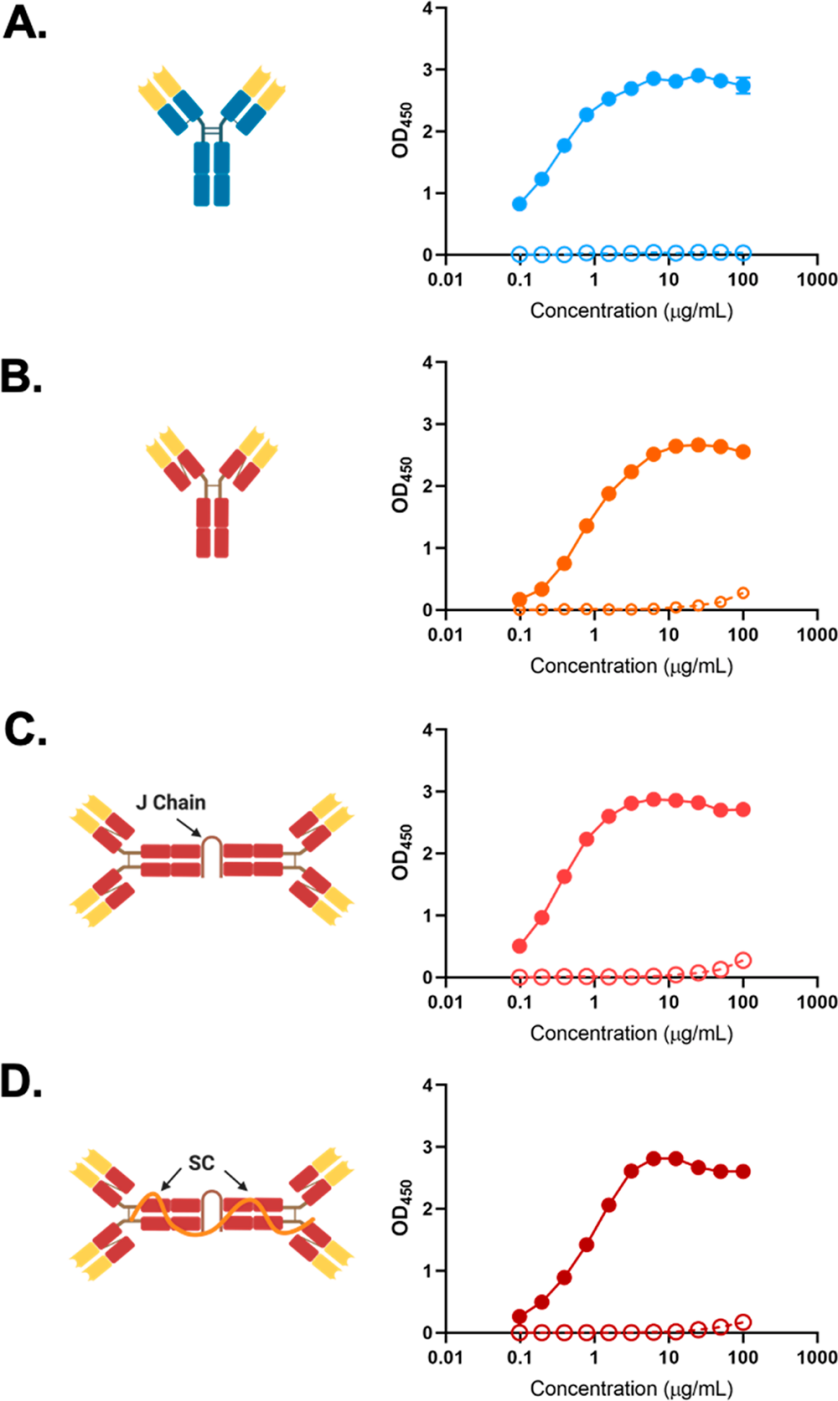
Sal4 mAbs bind STm lipopolysaccharide (LPS). Sal4 (A) IgG, (B) mIgA, (C) dIgA, and (D) SIgA binding to purified STm LPS as measured by ELISA. Filled circles represent Sal4 mAb reactivity, while empty circles represent isotype control antibody. ELISA graphs depict two technical replicates and are representative of two biological replicates.

As a first step in investigating the potential of recombinant human SIgA to protect against STm infection, we generated a chimeric form of Sal4 IgA in which the mouse V_H_ region was grafted onto a human IgA2 allotype m(2) backbone with the V_L_ element onto a human kappa light chain. Transient cotransfection of Expi293 cells with heavy and light chain constructs gave rise to monomeric IgA, as measured by size-exclusion chromatography (SEC) (Figure S1). Triple cotransfection with a plasmid encoding human J-chain resulted in the formation of IgA products that by SEC were consistent with dimer formation, while quadruple transfection with the addition of a vector encoding human SC resulted in the appearance of a product with a molecular weight of >280 kDa, consistent with the formation of SIgA. All three Sal4 IgA variants (mIgA, dIgA, SIgA) were affinity-purified as previously described.^29^ All forms of IgA bound to STm lipopolysaccharide (LPS) by ELISA, as detected with goat anti-human IgA secondary antibodies (Figure 1). Sal4 SIgA was specific for the O5-antigen, as demonstrated by ELISA (Figure S2).

**Figure 2:**
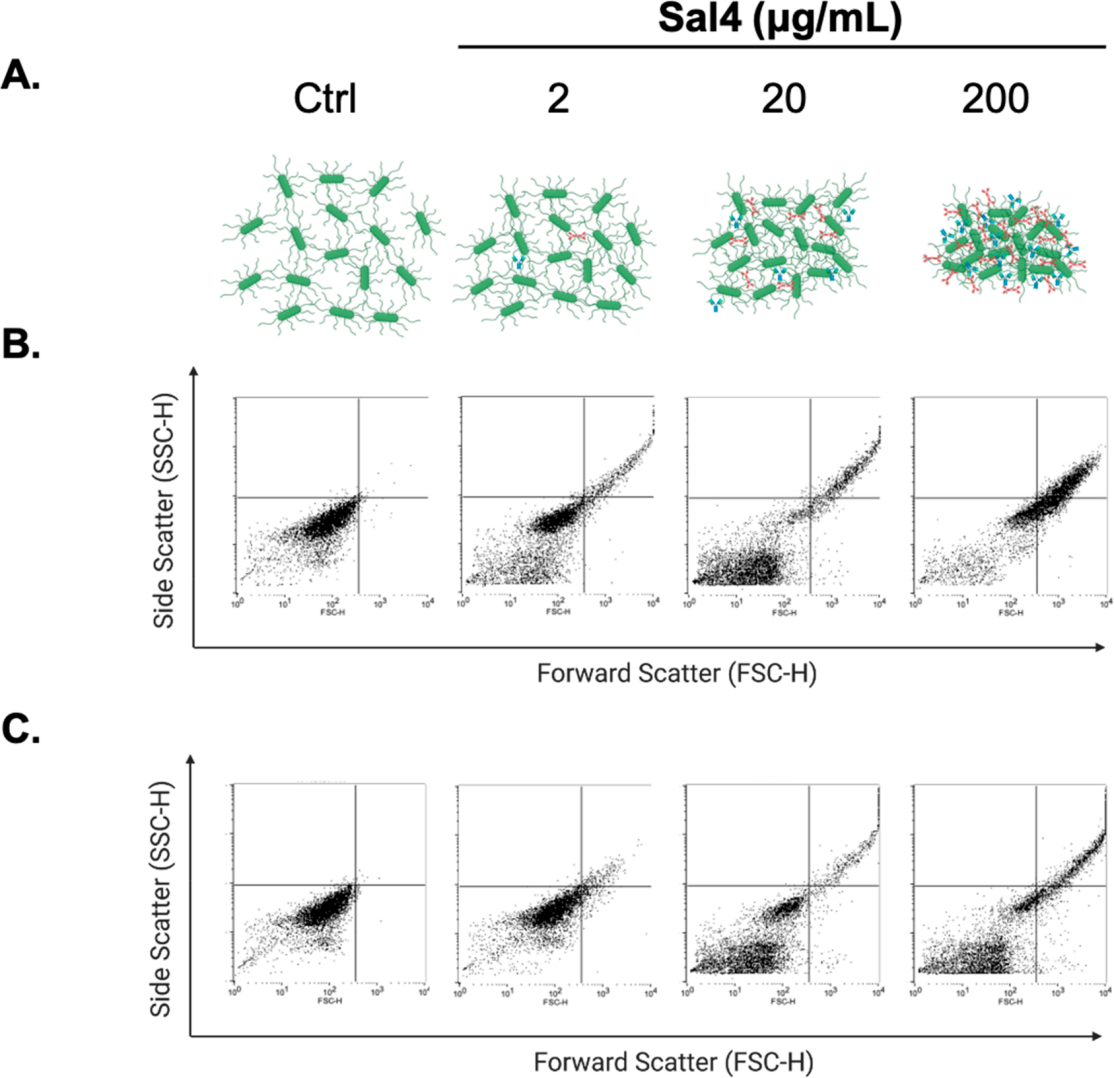
Sal4 mAbs agglutinate live STm cells by flow cytometry. Mid log phase cultures of AR05 were washed in PBS and incubated with 2, 20, or 200 μg/mL Sal4 SIgA or IgG or isotype control antibodies for 1 h at 37 °C. 10 000 events per sample were analyzed on a BD FACSCalibur (BD Biosciences, San Jose, CA) by forward scatter (FSC) and side scatter (SSC) to visualize aggregate size and granularity. Gating was set on untreated AR05 cells, and agglutination was defined by SSC-positive FSC-positive cells (Q2 + Q4), similar to that previously described.^38^ (A) Diagram demonstrating the antibody-to-bacterium ratio for each of the concentrations examined. (B, C) Representative flow cytometry plots showing FSC and SSC for (B) Sal4 SIgA and (C) Sal4 IgG and isotype control groups. STm cells treated with isotype control antibodies resulted in Q2 + Q4 values <1%. Results represent data from three separate biological experiments.

To further assess antibody functionality, Sal4 SIgA was examined by flow cytometry for the ability to induce bacterial agglutination.^38−40^ Mid log phase cultures of STm were treated with increasing amounts of Sal4 SIgA for 1 h at 37 °C and then analyzed by forward scatter (FSC) and side scatter (SSC) to quantify the size and frequency of STm–antibody complexes. We defined agglutination as the percentage of total events located in quadrants 2 (Q2) and 4 (Q4). Wild type STm strain AR05 cells treated with isotype control antibodies (or saline) had a maximal agglutination index of <1% (Table 1). Treatment of cells with Sal4 SIgA resulted in a dose-dependent increase in bacterial FSC and SSC that achieved an agglutination index of >65% in the presence of 200 μg/mL antibody (Table 1; Figure 2). The maximal agglutination index achieved with Sal4 IgG (200 μg/mL) was just ∼33% or roughly half of that observed for SIgA. It is notable that both Sal4 mIgA and dIgA variants were as effective as SIgA in promoting bacterial agglutination, as measured by flow cytometry (Table 1; Figure S3).

**Table 1:** Agglutination of STm Cells by Sal4 mAbs by Flow Cytometry

	% agglutination^a^ (mean ± SD)^b^		
	Sal4 conc.^c^ (μg/mL)		
	2	20	200
IgG	5.82 ± 4.50	19.19 ± 11.18	33.51 ± 14.81
mIgA	11.05 ± 3.39	31.46 ± 4.99	79.04 ± 2.46
dIgA	12.41 ± 5.20	38.85 ± 10.01	91.12 ± 3.07
SIgA	10.30 ± 2.78	23.39 ± 7.85	66.38 ± 4.98

a Gating was set on untreated AR05 cells, and agglutination was defined by SSC-positive FSC-positive cells (Q2 + Q4). STm cells treated with isotype control antibodies resulted in Q2 + Q4 values <1%.

b Results represent data from three separate biological experiments.

c Mid log phase AR05 cultures were washed in PBS and incubated with indicated amounts of Sal4 mAbs for 1 h at 37 °C. 10 000 events per sample were analyzed on a BD FACSCalibur (BD Biosciences, San Jose, CA) by forward scatter (FSC) and side scatter (SSC) to visualize aggregate size and granularity.

**Figure 3:**
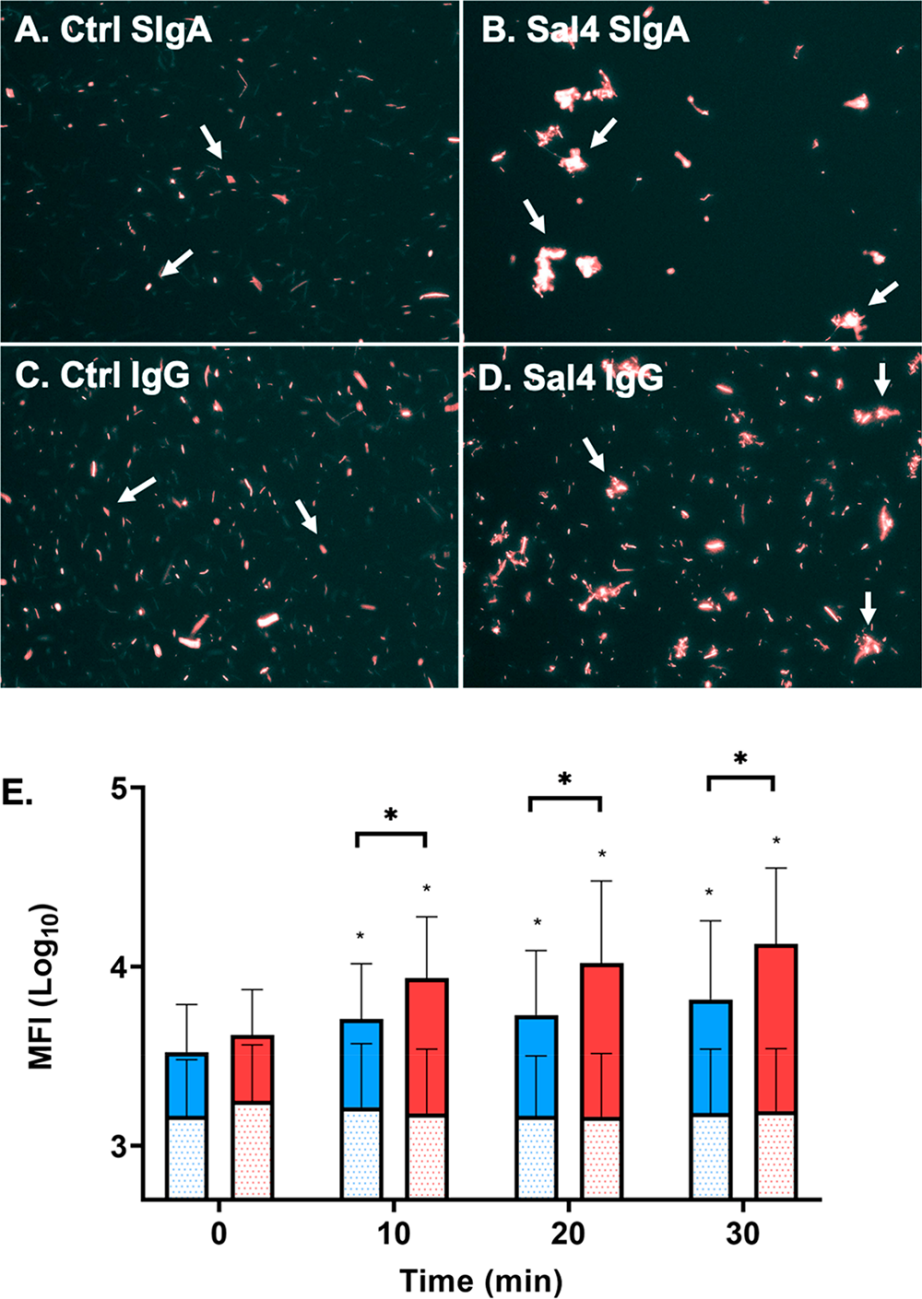
Sal4 SIgA significantly agglutinates mCherry-expressing STm cells. Mid log phase AR05-mCherry cells were induced in 0.2% arabinose and treated with 15 μg/mL (A) control IgA, (B) Sal4 SIgA, (C) control IgG, or (D) Sal4 IgG at room temperature. Cells were spotted on uncharged microscope slides at 10 min intervals for 30 min. Four to 7 images were taken at 20× for each condition and time point in the Texas Red channel. Images were analyzed for mean fluorescence intensity (MFI) per aggregate using Fiji as described in the Methods. Arrows indicate primarily single cells present in the control groups compared to bacterial aggregates in Sal4 treatment groups. (E) Quantification of mean MFI for Sal4 SIgA (red) and Sal4 IgG (blue) treatment groups. Isotype control values for each antibody are shown as shaded bars. Data represents three biological replicate experiments. Statistical significance was determined by one-way ANOVA followed by Tukey’s post hoc multiple comparisons test. Asterisks (*) on the bars indicate *p* < 0.05 compared to the isotype control. *p < 0.05 between treatment groups.

We next used fluorescence microscopy to capture STm agglutination in real-time. We used a strain of STm AR05 expressing mCherry under the control of an arabinose-inducible promoter. STm cells were grown to mid log phase in the presence of 0.2% arabinose, treated with antibody, and then spotted on microscope slides. Four to seven images were taken at 20× magnification, and the mean fluorescence intensity (MFI) of STm–antibody complexes was measured over time. Bacteria treated with an isotype control antibody remained motile and uniformly dispersed within the liquid medium. There was no evidence of aggregation at any time point among the control groups (Figure 3A,C). When treated with Sal4 IgG, STm formed granular aggregates within 10 min (Figure 3D,E). The MFI of STm–Sal4 IgG aggregates at 10 min was ∼3400 and by 30 min reached ∼5000 (Figure 3E). Sal4 SIgA was even more efficient at inducing bacterial aggregation, as evidenced by an MFI of ∼7000 at 10 min and ∼11 000 by 30 min (Figure 3B,E). The higher MFI values associated with the Sal4 SIgA treatment were the result of the formation of both larger and more dense aggregates than those observed by Sal4 IgG.

### Sal4 SIgA Inhibits STm Invasion of Peyer’s Patch Tissues in a Mouse Model

**Figure 4:**
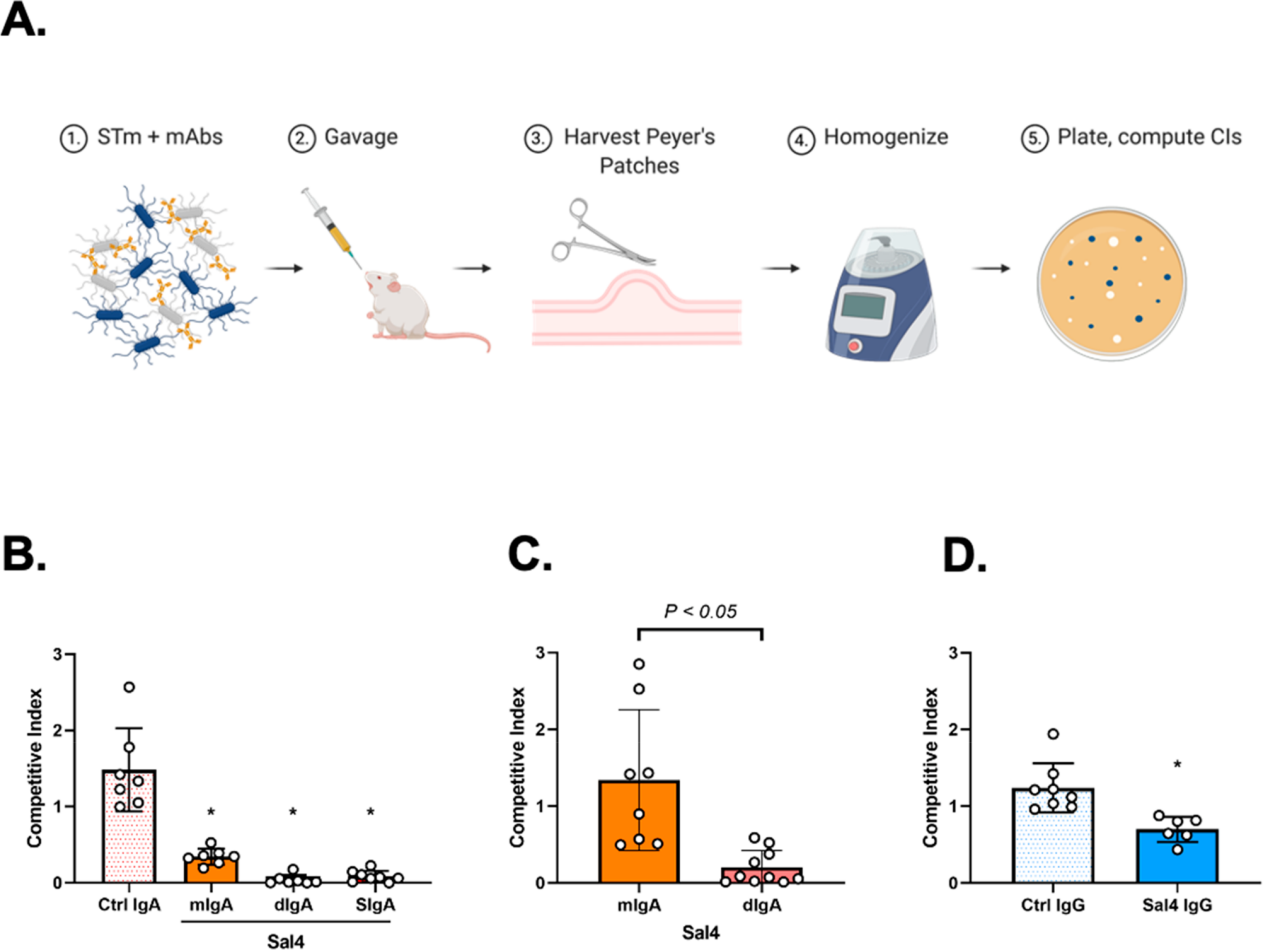
Sal4 SIgA blocks STm entry into Peyer’s patches. (A) Schematic of the STm infection model. BALB/c female mice are orally administered Sal4 or isotype control antibody in PBS immediately prior to a 1:1 mixture of AR04 and AR05 STm strains (∼4 × 10^7^ CFUs). Twenty-four hours later mice are sacrificed, and a laparotomy is performed to isolate Peyer’s patches from the small intestine. Peyer’s patches from each mouse are pooled in 1 mL of ice-cold sterile PBS, homogenized, and plated for CFUs on LB agar containing kanamycin and X-gal. (B–D) STm invasion into Peyer’s patches of mice treated with (B) 50 μg of Sal4 mIgA, dIgA, or SIgA, (C) 10 μg of Sal4 mIgA or dIgA, or (D) 50 μg of IgG at the time of the STm challenge. Shown are the combined results of two separate experiments with at least 4 mice per group. Statistical significance was assessed by one-way ANOVA followed by Tukey’s post hoc multiple comparisons test.

We next evaluated Sal4 SIgA for the ability to block STm invasion into Peyer’s patch tissues, which represent the primary portal of entry for STm in the intestinal mucosa.^41,42^ As shown in Figure 4A, BALB/c mice were gavaged with a 1-to-1 mixture (4 × 10^7^ CFUs per mouse) of kanamycin-resistant STm strains AR05 and AR04. AR04 is a derivative of AR05 that constitutively expresses β-galactosidase and lacks the O5 epitope due to a transposon insertion within *oafA*.^43^ As such, AR04 does not react with Sal4 and serves as an internal control.^36^ Mice were euthanized ∼24 h later, and Peyer’s patch tissues were removed and homogenized. The homogenates were plated onto LB agar containing kanamycin and the chromogenic substrate 5-bromo-4-chloro-3-indolyl-β-d-galactopyranoside (X-Gal) to differentiate AR04 (LacZ^+^) from AR05 (LacZ^–^) by blue-white screening. The ratio of AR05 to AR04 in the challenge dose (input) was compared to the ratio of AR05 to AR04 in Peyer’s patch lysates (output) to yield a competitive index (CI).

In the mouse model, Sal4 SIgA reduced AR05 invasion of Peyer’s patch tissues by several orders of magnitude, as demonstrated by a CI value of 0.09 ± 0.07. The same concentration of Sal4 mIgA and dIgA variants also significantly inhibited AR05 infection, as evidenced by CI values of 0.34 and 0.04, respectively (Figure 4B). At lower antibody doses, dIgA was slightly more effective than mIgA, revealing the contribution of antibody valency in limiting bacterial entry into Peyer’s patch tissues (Figure 4C). Sal4 IgG, in contrast, only marginally influenced STm AR05 infection, as evidenced by a CI of 0.7 ± 0.164 (Figure 4D). This result agrees with a previous study in which even >250 μg of Sal4 IgG had no significant impact on STm entry into Peyer’s patches.^36^

To examine whether Sal4 SIgA can function prophylactically, Sal4 SIgA (50 μg per animal) was administered to mice by gavage at 40, 20, or 1 min prior to the STm challenge. When administered immediately prior to the bacterial challenge, Sal4 SIgA significantly reduced AR05 entry into Peyer’s patch tissues (Figure S4). However, the administration of Sal4 SIgA at 40 or 20 min prior to the bacterial challenge did not have any appreciable effect on the AR05 infection. Co-administration of SIgA with sodium bicarbonate (to buffer gastric pH) and protease inhibitors (to neutralize gastric and intestinal proteases) did not significantly improve Sal4 SIgA prophylactic activity (Figure S4), suggesting that antibody degradation was not a limiting factor. Collectively, we conclude from these studies that recombinant human Sal4 SIgA, Sal4 dIgA, and to some degree mIgA inhibit the earliest steps in STm infection of gut-associated lymphoid tissues, possibly due to bacterial agglutination and entrapment in the intestinal lumen.

### Recombinant Human Sal4 SIgA Promotes STm Agglutination in the Intestinal Lumen

Several lines of evidence suggest that Sal4 SIgA-mediated agglutination of STm is qualitatively and quantitatively different than agglutination induced by Sal4 IgG. For example, by flow cytometry, Sal4 SIgA induced an agglutination index that was approximately twice that of Sal4 IgG (Table 1). In macro-agglutination assays performed in microtiter wells, Sal4 SIgA (but not Sal4 IgG) promoted the formation of bacterial “mats” that were impervious to vigorous pipetting.^36^ Finally, scanning electron microscopy (SEM) analysis of bacterial aggregates induced by mouse Sal4 IgA hybridoma supernatants revealed gross alterations in cell morphology, especially at points of cell–cell contact.^44^

**Figure 5:**
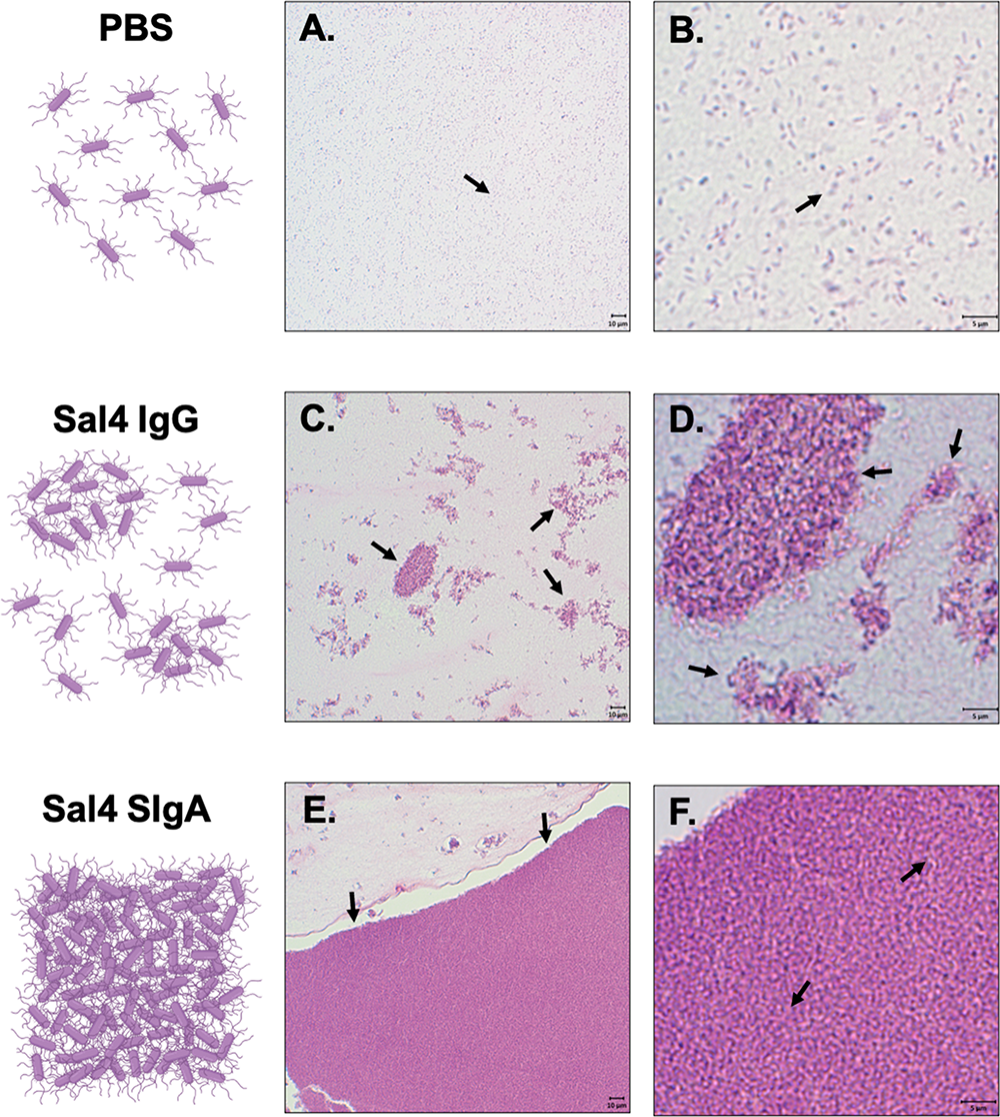
H&E staining of STm agglutination by Sal4 SIgA and IgG. Mid log phase ATCC14028 STm cells were treated with (A, B) PBS or 50 μg of (C, D) Sal4 IgG or (E, F) Sal4 SIgA, pelleted, and immobilized in bovine–thrombin plasma clots as described in the Methods. Clots were fixed and prepared for paraffin-embedding and sectioning. Samples were counterstained with H&E to visualize STm–antibody aggregates. (A, B) STm cells in PBS were uniformly dispersed as single cells. (C, D) Sal4 IgG generated numerous clusters of STm bacteria primarily arranged in lacy and irregular patterns that appeared loosely packed. (E, F) Sal4 SIgA treatment induced large sheets of tightly packed bacteria that spanned the field of view on the microscope. Single cells were rarely observed in SIgA-treated cells compared to PBS and IgG groups. Scale bars depict 10 μm (low magnification) and 5 μm (high magnification) as indicated.

To visualize the nature of the bacterial aggregates induced by recombinant human Sal4 SIgA, mid log phase STm cultures were treated with 50 μg of Sal4 IgG or SIgA, collected by centrifugation, and then entrapped in bovine thrombin–plasma clots as already described.^45^ The clots were embedded in paraffin, sectioned, and counterstained with H&E to further distinguish STm–antibody complexes. Saline-treated STm cells were uniformly dispersed within the fields of view across multiple sections of the clots (Figure 5A,B). Sal4 IgG-treated STm cells were clustered in small, loosely packed aggregates that had a lacy appearance (Figure 5C,D). In contrast, Sal4 SIgA-treated cells formed large masses that encompassed entire fields of view under low magnification (Figure 5E,F). Closer inspection indicated that the cells were densely packed to the point where individual bacteria were not readily discernible.

To determine whether similar aggregates occur *in vivo*, we gavaged mice with Sal4 SIgA, Sal4 IgG, or vehicle alone (PBS) followed immediately by a bolus of STm (∼4 × 10^7^ CFUs). The mice were euthanized 20 or 40 min later, and the length of the GI tract from the pyloric sphincter to the rectum was excised, fixed in paraformaldehyde, embedded in paraffin blocks, and sectioned for immunohistochemistry (IHC) as described in the Methods. Tissue sections were deparaffinized, rehydrated, and subjected to antigen retrieval by proteinase K incubation. To visualize STm *in situ*, paraffin sections were stained with rabbit *Salmonella* Group B-specific antisera followed by alkaline phosphatase (AP)-polymer and chromogen application.

**Figure 6:**
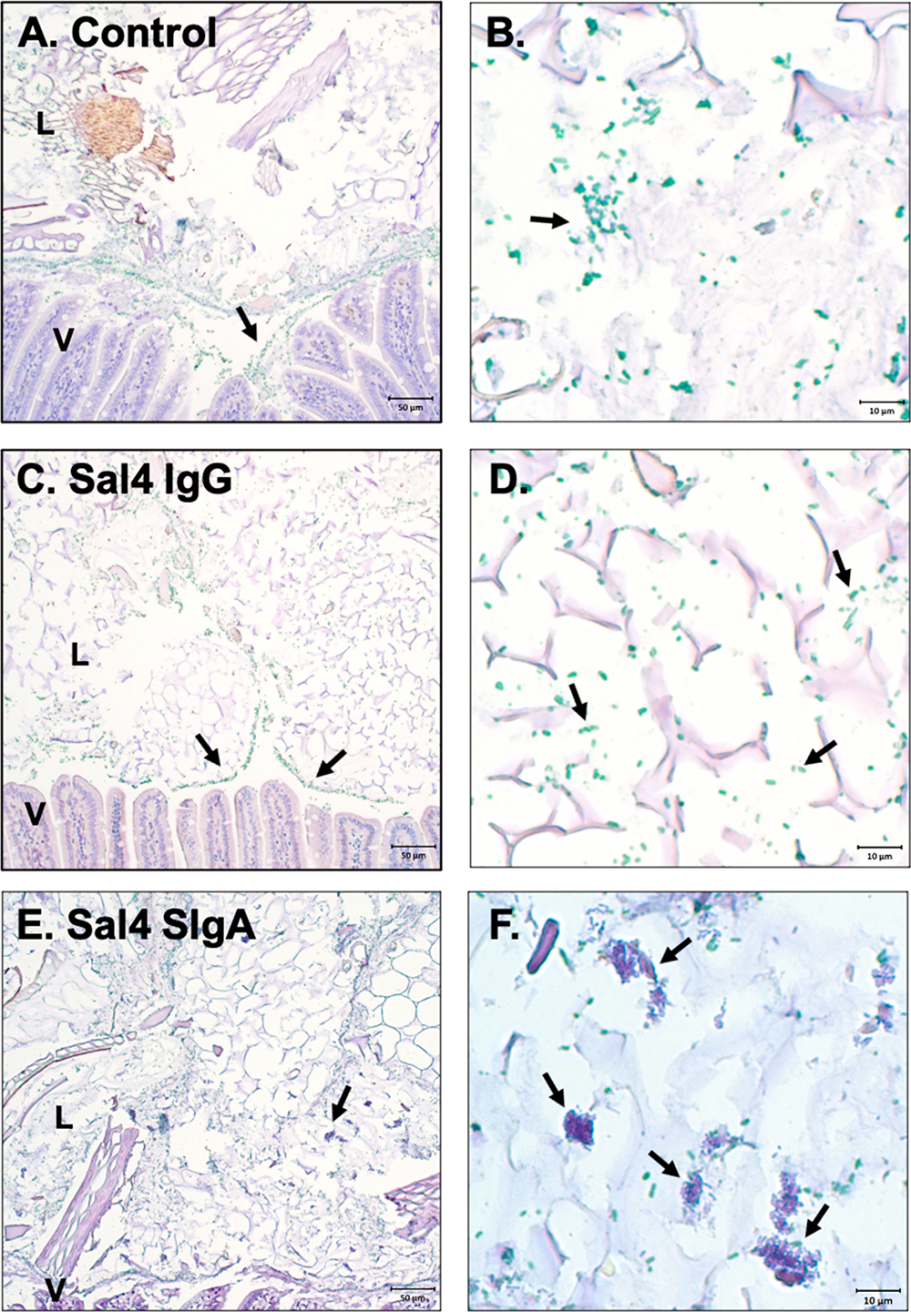
Sal4 SIgA agglutinates STm cells in the intestines of infected mice at 40 min. BALB/c female mice were orally administered (A, B) PBS or 50 μg of (C, D) Sal4 IgG or (E, F) Sal4 SIgA prior to ∼4 × 10^7^ CFUs of STm wild type strain ATCC14028. Animals were euthanized 40 min postinfection, and the mouse gastrointestinal tracts were isolated, fixed, and prepared for immunohistochemistry. Tissue samples were subjected to antigen retrieval via proteinase K incubation, and STm cells were stained using rabbit Salmonella Group B-specific antiserum (BD Difco) and AP-polymer and chromogen application. Positively stained STm cells are depicted in green and were predominately stained in tissue samples from PBS and Sal4 IgG-treated animals (A–D). STm cells in Sal4 SIgA-treated animals were found in large aggregates in the intestinal lumen with only a portion of cells positively stained by IHC (E, F). Positive cells in Sal4 SIgA-treated animals were primarily at the periphery of the STm aggregates. Scale bars depict 50 μm (low magnification) and 10 μm (high magnification) as indicated.

STm was localized to the jejunum and upper portion of the ileum in tissues collected at 20 min (data not shown). After 40 min, STm was detected in the distal ileum and cecum (Figure 6). Bacteria were interspersed in the intestinal lumen with occasional cells sequestered in the mucus layer. Most of the inoculum, however, was present as single cells or in small clusters (Figure 6A,B). The pattern was similar in mice treated Sal4 IgG. STm cells were uniformly distributed in the lumen and occasionally found in small, loosely packed clusters in the terminal ileum and cecum (Figure 6C,D). In contrast, in Sal4 SIgA-treated mice, STm appeared in large, dense clusters in the ileum and cecum (Figure 6E,F). The STm aggregates were refractory to IHC with anti-LPS polyclonal antibody, as evidenced by Vina Green Chromogen staining around the periphery but not in the center of the cell clusters (Figures 6E,F and S5). Even aggressive antigen retrieval methods such as heat-induced epitope retrieval with citrate buffer followed by proteinase K treatment did not render the clusters accessible to antibody staining (data not shown). These results are consistent with Sal4 SIgA promoting cell–cell cross-linking *in vitro* and *in vivo* that likely renders STm incapable of accessing the Peyer’s patch tissues.

### STm Extracellular Matrix (ECM) Production Following Sal4 SIgA Treatment

**Figure 7:**
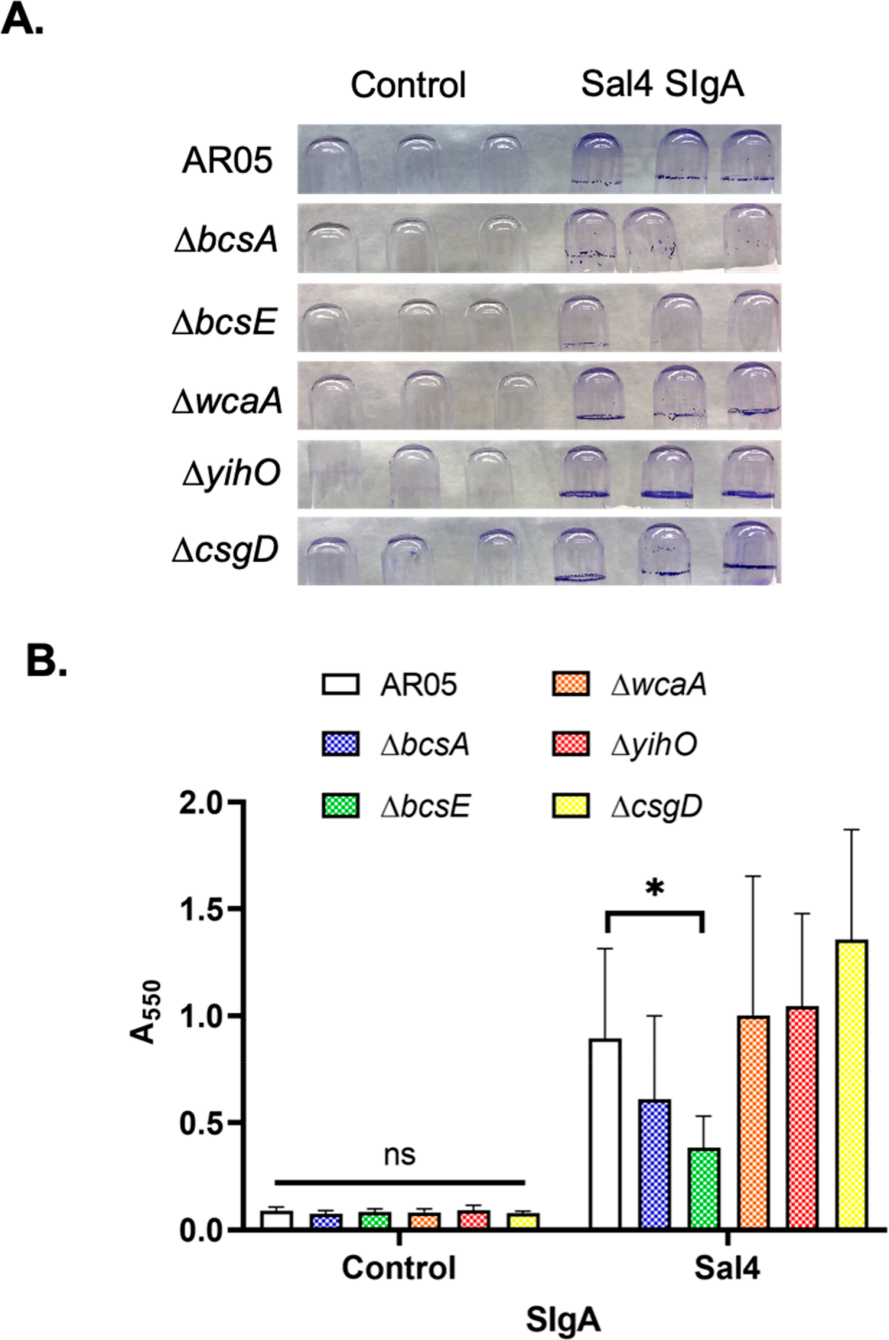
CV staining of biofilm mutants in response to Sal4 SIgA. STm wild type strain AR05 and biofilm mutant strains Δ*bcsA* and Δ*bcsE* (cellulose), Δ*wcaA* (colanic acid), Δ*yihO* (O-Antigen capsule), and Δ*csgD* (biofilm regulator) were grown to mid log phase and incubated in borosilicate glass tubes with 50 μg/mL Sal4 SIgA or control SIgA for 1 h at 37 °C under shaking conditions. Tubes were washed in PBS, fixed with methanol, and stained with 0.1% crystal violet (CV). (A) Representative images depict CV staining following antibody treatment. (B) Solubilized CV was quantified at *A*_550_. Data represents three biological replicates each performed with three technical replicates. Statistical significance was determined by two-way ANOVA followed by Tukey’s post hoc multiple comparison test. *p < 0.05.

The recalcitrant nature of the STm aggregates induced by Sal4 SIgA are reminiscent of extracellular matrix (ECM)-encased bacterial mats induced during in the early stages of bacterial biofilm formation.^46−48^ Indeed, we have reported that mouse Sal4 IgA stimulates STm to secrete an ECM containing (but not limited to) cellulose, colanic acid, and O-antigen.^49^ To examine whether human Sal4 SIgA triggers STm to secrete ECM, mid log phase cultures of AR05 grown in borosilicate glass tubes with aeration were treated with Sal4 SIgA (50 μg/mL) or an isotype control for 1 h at 37 °C and then subjected to crystal violet (CV) staining. As compared to controls, Sal4 SIgA-treated cells secreted copious amounts of ECM, particularly at the air–liquid interface (Figure 7A,B). We employed available STm strains with mutations in cellulose biosynthesis (Δ*bcsA*, Δ*bcsE*),^50,51^ colanic acid production (Δ*wcaA*), and O-antigen capsule formation (Δ*yihO*)^52^ to dissect which (if any) of these components contributed to the CV staining observed following Sal4 SIgA treatment. We also examined the role of CsgD, a known transcriptional regulator of *Salmonella* biofilm formation, in ECM expression in response to Sal4 SIgA treatment.^53^ While the STm Δ*wcaA*, Δ*yihO*, and Δ*csgD* mutants produced CV levels similar to the wild type strain (Figure 7B), the STm Δ*bcsA* and Δ*bcsE* mutants each secreted less ECM than the control (Figure 7B). The phenotype was more pronounced with the Δ*bcsE* mutant than the Δ*bcsA* mutant, which is interesting considering that BcsE binds c-di-GMP and is required for optimal cellulose production.^51^ In preliminary studies, we found that the treatment of STm by Sal4 IgG also stimulated CV activity. However, the ECM profiles between Sal4 SIgA and Sal4 IgG were different in that SIgA triggered cellulose-dependent ECM, while IgG induced cellulose-independent ECM (data not shown).

Our results suggest that Sal4 SIgA promotes the formation of STm aggregates encased in cellulose and possibly other ECM components, thereby rendering the bacteria entrapped in the intestinal lumen and unable to penetrate Peyer’s patch tissues. If cellulose was limiting bacterial entry into the Peyer’s patch tissues, then we predicted that the *bscA* or *bcsE* mutant would be more invasive than the wild type strain in Peyer’s patch tissues in the presence of Sal4 SIgA. To test this hypothesis, the *bcsA* and *bcsE* mutants were tested for Peyer’s patch invasion in the mouse model in the absence and presence of Sal4 SIgA. We found that Sal4 inhibited the invasion of the STm *bscA* or *bcsE* mutants to a similar degree as the wild type strain, indicating that the cellulose mutants did not “escape” the effects of Sal4 SIgA (data not shown). We speculate that cellulose secretion may play a role in other aspects of STm pathogenesis aside from invasion, such as survivability within the gut or host transmission.

## Discussion

In this study, we produced and characterized a recombinant human SIgA form of the monoclonal antibody, Sal4. Sal4 was originally isolated from a B cell hybridoma generated from mice that had been orally immunized with attenuated strains of STm. Sal4 IgA is specific for the immunodominant O5-antigen of STm LPS and is capable of preventing the invasion of polarized epithelial monolayers by virulent STm. In mice, the delivery of Sal4 IgA via the “backpack tumor” model or by gavage is sufficient to limit bacterial uptake into Peyer’s patch tissues.^34,36^ It is also reported that Sal4 IgA treatment inhibits STm motility and abrogates the STm SPI-1 type III secretion system (T3SS), each of which contribute to bacterial entry into the epithelial cells.^54^ The motivation behind our current endeavor was to investigate whether a recombinant human SIgA form of Sal4 retains biological activity and is able to block bacterial entry into gut associated-lymphoid tissues when administered passively.

Historically, recombinant SIgA has been difficult to produce because of the complex nature of the molecule.^55^ Corthésy and colleagues were one of the few teams who successfully employed SEC to purify mIgA, dIgA, and pIgA from B cell hybridoma supernatants.^56^ They were able to reconstitute SIgA *in vitro* by complexing dIgA and pIgA with recombinant SC.^27^ Other groups have expressed recombinant SIgA in transgenic plants,^57^ but only recently have mammalian cell-based strategies proven fruitful.^29,30,37^ Moreover, the structure of SIgA has been revealed through X-ray and cryo-EM techniques.^11,16,22^ By all accounts, the recombinant human Sal4 SIgA produced in our study has potent biological activity *in vitro* and *in vivo*.

Our results are consistent with immune exclusion as being the primary mechanism by which recombinant human SIgA Sal4 limits STm uptake into mouse Peyer’s patch tissues. In our studies, large and densely packed aggregates of STm were evident in the intestinal lumen of mice that had been pretreated with Sal4 SIgA. In contrast, there was no such evidence for STm aggregation in mice treated with Sal4 IgG. Rather, in those mice, STm was generally observed as individual cells throughout the lumen and dispersed within the mucosa. These observations are essentially in accordance with SIgA (but not IgG) being the primary mediator of immune exclusion.^19^ However, we acknowledge that there is certainly more to the story. As noted above, we have reported that Sal4 IgA inhibits STm’s flagella-based motility by a mechanism that may involve membrane depolarization and/or dinucleotide signaling cascades.^36,49,54,58^ Moor et al. reported in a mouse model that high avidity polyclonal IgA elicited by mucosal vaccination of STm promotes enchained bacterial growth within the gut.^39^ Enchained growth is proposed to primarily act in low-density bacterial environments where agglutination with neighboring cells is infrequent.

Nonetheless, one caveat worth noting is that SIgA given via gavage is unlikely to assume the normal distribution pattern of SIgA that is transported into the gut via pIgR. In effect, the pIgR-mediated delivery of SIgA into the gut results in an “inside-to-outside” diffusion pattern, whereas oral delivery would be subjected to the opposite (“outside-to-inside”). Moreover, orally delivered SIgA is subject to different transit times through the gastrointestinal tract and is likely diluted significantly within minutes after being deposited into the stomach.^59^ Collectively, these factors may account for why Sal4 SIgA pretreatment at −20 and −40 min was largely ineffective at blocking STm infectivity in our model.

On the basis of the results of our current study, we postulate that STm, upon exposure to Sal4 SIgA, becomes encased in a bacterium-derived ECM that is reminiscent of early biofilm formation. As described by Gunn et al., bacteria like STm produce complex extracellular matrices consisting of myriad exopolysaccharides, extracellular DNA, and proteins (e.g., amyloids, flagella).^60^ In the case of STm, the major ECM components include cellulose, colanic acid, and an O-antigen capsule. Using STm mutants deficient in the synthesis of these substances, we identified cellulose as the likely candidate contributing to ECM production in response to Sal4 SIgA. This is consistent with previous findings from our laboratory.^49^ We can only speculate that the secretion of ECM by STm in response to Sal4 SIgA is a defensive mechanism by which the bacteria render themselves recalcitrant to further attack by other components of the mucosal immune system.^60^ We are currently investigating whether STm “senses” an antibody attack through a signaling pathway involving a cyclic diguanylate monophosphate (c-di-GMP) known to regulate motility and cellulose production.^46,49,50,58,61^

Ultimately, our study highlights the opportunities and formidable challenges associated with passive oral immunization with recombinant human SIgA. In the literature, orally delivered antibodies have been demonstrated to protect against diarrheal pathogens, such as ETEC.^62^ A similar prophylactic potential has been reported by pooled plasma-derived antibodies to alleviate intestinal inflammation following mucosal STm infection in mice.^63^ However, these studies, as well as ours, underscore that adequate dosing is a major barrier to the use of SIgA prophylactically. As noted earlier, we speculate that the protection against the STm invasion of the Peyer’s patch tissues is only achieved when Sal4 SIgA levels exceed a critical threshold concentration required to promote bacterial agglutination. Indeed, in a controlled human infection model, multiple doses of hyperimmune bovine colostrum (HBC) before and after the oral ETEC challenge were necessary to prevent diarrhea.^64^ In mice, preincubation of the ETEC with SIgA mAbs was required to reduce bacterial colonization.^37^ Similar issues with achieving sufficient local antibody concentrations in the gut apply in the pursuit of using oral mAbs for therapeutic use for STm. While, in a mouse model, human plasma-derived secretory antibodies have been reported to promote survival after the STm challenge, the window of protection was limited to 8 h after the initial inoculation, and a number of the animals still succumbed to infection at the end of the study.^35^ Despite limitations, these results emphasize the developing potential of recombinant SIgA as an alternative approach for combatting enteric pathogens.

## Methods

### Monoclonal Antibodies (mAbs)

Antibodies used in this study are listed in Table 2. Sal4 mIgA, dIgA, and SIgA mAbs were generated as described previously.^29^ In brief, Expi293 cells were transiently transfected with a construct containing the sequence for the Sal4 heavy chain variable domain (V_H_) in a human IgA2 allotype m(2) alongside the corresponding Sal4 light chain (V_L_). Sal4 dIgA and SIgA were cotransfected with a plasmid containing joining (J)-chain with Sal4 SIgA additionally expressed with a secretory component (SC). Supernatants were collected and purified using CaptureSelect IgA Affinity Matrix (Thermo Fisher). Chimeric Sal4 hIgG1 and HD9-N IgG1, specific for ricin toxin, were provided by Mapp Biopharmaceutical (San Diego, CA). Human IgA from colostrum was obtained from Sigma (St. Louis, MO).

**Table 2:** Antibodies and mAbs Used in This Study

antibody	species	isoform	source	epitope
Sal4	human	mIgA2 m(2)	Expi293F cells	STm O5-LPS
Sal4	human	dIgA2 m(2)	Expi293F cells	STm O5-LPS
Sal4	human	SIgA2 m(2)	Expi293F cells	STm O5-LPS
Sal4	chimeric	IgG1	*Nicotiana benthamiana*	STm O5-LPS
HD9-N	humanized	IgG1	*Nicotiana benthamiana*	ricin toxin
IgA (colostrum)	human	SIgA	Sigma (#I2636)	polyclonal

### Size-Exclusion Chromatography Ultra High-Performance Liquid Chromatography (SEC-UHPLC)

Size-exclusion chromatography was performed with a series of columns consisting of an Acquity UPLC Protein BEH SEC Guard Column (WAT186006850), and Acquity UPLC Protein BEH SEC, 200 A (WAT176003904, 1.7 μm, 4.6 × 150), and Acquity UPLC Protein BEH SEC, 450 A (WAT176002996, 2.5 μm, 4.6 × 150 mm) using an Agilent 1260 Infinity Quaternary Bioinert LC. Purified mAbs were filtered with a 0.22 μm filter and centrifuged for 5 min at 10 000*g* before 5 μL of each sample was injected in the columns at 30 °C with a flow rate of 0.35 mL/min in PBS + 350 mM NaCl, pH 6.9. Protein detection was performed at 280 nm. A BEH200 SEC Protein Standard Mix (Waters 186006518) was used to benchmark the samples’ molecular weights.

### Enzyme-Linked Immunosorbent Assays (ELISAs)

For STm-LPS ELISAs, Immulon Microtiter 96-well plates (ThermoScientific, Waltham, MA) were coated with 100 μL/well of purified STm-LPS (#L6143, Sigma-Aldrich) in sterile PBS overnight at 4 °C. Plates were washed three times in PBS containing 0.1% Tween-20 (PBS-T) and blocked at room temperature for 2 h in 200 μL/well PBS-T containing 2% goat serum. After blocking, primary antibodies were diluted in block and incubated for 1 h. Plates were washed four times in PBS-T and incubated with either goat-antihuman IgG-HRP or goat-antihuman IgA-HRP secondary antibodies (final concentration of 0.5 μg/mL) for 1 h. Plates were washed in PBS-T four times prior to being developed with 100 μL/well SureBlue TMB 1-Component Microwell Peroxidase Substrate (SeraCare, Milford, MA). For whole-cell ELISAs, Immulon Microtiter 96-well plates were coated with 50 μL/well poly-l-lysine (10 μg/mL) overnight at 4 °C. Mid log phase cultures (OD_600_ of 0.7) of STm strains were washed twice with sterile PBS, applied to coated plates, and centrifuged at 1500*g* for 6 min, rotating the plate 180° halfway through the spin. Plates were incubated with 16% paraformaldehyde for 15 min, washed in PBS-T, and incubated in fresh 0.1 M glycine for 30 additional minutes. Plates were washed a final time before blocking overnight in PBS-T + 2% goat serum. Whole-cell ELISAs were performed the following day as described above. All plates were read using a VersaMax spectrophotometry microplate reader at 450 nm absorbance (*A*_450_) using SoftMaxPro 5.2 software.

### Bacterial Strains and Growth Conditions

*Salmonella* Typhimurium strains used in this study are listed in Table 3. All *S.* Typhimurium strains are derivatives from the original American Type Culture Collection (ATCC) STm isolate 14028 (Manassas, VA). AR04 and AR05 have been described in detail previously.^36,54,58^ AR05-mCherry contains an arabinose-inducible plasmid under gentamicin antibiotic pressure (10 μg/mL) that allows for the expression of mCherry fluorescent protein by AR05 cells in the presence of arabinose (0.2% final concentration). Unless otherwise stated, overnight cultures were inoculated with a single bacterial colony in 5 mL of Luria–Bertani (LB) broth and incubated overnight (∼16 h) at 37 °C with aeration at 200 rpm. Overnight cultures were subcultured to upper mid log phase (OD_600_ of 0.7) prior to experimentation.

**Table 3:** STm Strains Used in This Study

	O–Ag	genotype	reference
strain			
ATCC14028	O5	wild type	(71)
AR05	O5	*zjg8101::kan*	(58)
AR04	O4	*zjg8101::kan oafA126::*Tn*10d*-Tc *fkpA-lacZ*	(58)
AR05-mCherry	O5	*zjg8101::kan*; *pMW232*	this study
Δ*bcsA*	O5	Δ*bcsA*	this study
Δ*bcsE*	O5	*bscE::kan*; kanamycin cassette insertion mutation in *bscE* gene	(49)
Δ*csgD*	O5	deletion mutant of *csgD*	(49)
Δ*wcaA*	O5	*wcaA::luc*; a mutant of *wcaA* constructed using luciferase-reporter suicide vector pGPL01	(72)
Δ*yihO*	O5	λ red deletion of *yihO*	(73)
plasmid			
pMQ80		high copy *Pseudomonas* vector harboring *araC* and *aacC1* (gentR)	(65)
pUCIDT-KAN-mCherry_Pa_		*mCherry*_*Pa*_ in pUCIDT-KAN	this study
pMW232		*P*_*BAD*_*-mCherry*_*Pa*_ in pMQ80	this study

### Generation of mCherry-Expressing STm Strains

A high copy plasmid harboring an arabinose-inducible mCherry cassette (pMW232) was engineered for expression in *Salmonella* Typhimurium. pMW232 was created by replacing the arabinose-inducible *GFPmut3* ORF in pMQ80^65^ with a codon optimized variant of *mCherry* designed for expression in *Pseudomonas aeruginosa*. To accomplish this, pMQ80 was first subjected to restriction digestion with *Kpn*I and *Hin*dIII (NEB) to remove the *GFPmut3* ORF and associated Shine-Dalgarno sequence. The *mCherry* ORF was then amplified from pIDTSmart-Kan-mCherry_Pa_ (Integrated DNA Technologies) using Q5 DNA polymerase (NEB) and tailed primers (mcherry_F_SD_*Kpn*I, 5′-TCGGTACCCGGAGAAGGAGATATACATATGGTGAGCAAGGGCGAGGAGGA-3′; mcherry_R_*Hin*dIII, 5′-CAGAAGCTTCTACTTGTACAGCTCGTCCATGCCG-3′) that were designed to incorporate a 5′ *E. coli* Shine-Dalgarno consensus sequence and flanking *Kpn*I and *Hin*dIII restriction sites. The amplified DNA fragment was then digested with *Kpn*I and *Hin*dIII, ligated into similarly cut pMQ80, transformed into chemically competent NEB 5 alpha cells (NEB), plated on LB agar supplemented with 10 μg/mL gentamicin, and then incubated overnight at 37 °C. Gentamicin-resistant colonies that emerged were subsequently screened for the incorporation of the *mCherry* ORF via culturing in LB supplemented with 10 μg/mL gentamicin and 0.2% l-arabinose. pMW232 was then harvested from a culture that turned pink after several hours of growth using a Qiagen’s Miniprep kit and the provided protocol. Electrocompetent *Salmonella* Typhimurium (AR05) cells were then prepared and transformed with pMW232 using previously described methodology to generate the AR05-mCherry strain.^66^

### Agglutination by Flow Cytometry

AR05 cultures were subcultured to mid log phase, washed twice with sterile PBS by pelleting cells at 6000*g* for 3 min, resuspended in PBS. STm cells were incubated with the indicated antibody treatments for 1 h at 37 °C before being transferred to round-bottom polystyrene tubes for flow cytometry. Live samples were analyzed on a BD FACSCalibur (BD Biosciences, San Jose, CA) similar to that described in ref (38). STm groups were gated in forward scatter (FSC) and side scatter (SSC) to visualize cell aggregate size and granularity. Untreated AR05 was used for thresholding and gating. Agglutination was calculated by adding FSC-positive and SSC-positive quadrants (Q2 + Q4) as already described.^67^ 10 000 events were acquired per sample using CellQuest Pro (BD Biosciences), and samples were analyzed in three biological replicates using GraphPad Prism 8 (San Diego, CA).

### Agglutination by Fluorescence Microscopy

AR05-mCherry overnight cultures were inoculated in LB broth containing 10 μg/mL gentamicin and 0.2% sterile arabinose and were incubated with aeration at 37 °C. Cells were subcultured 1:50 in the same media until an OD_600_ of ∼0.5 was reached. Fluorescent STm samples were examined visually to confirm proper fluorescence intensity and cell morphology at the beginning of each experiment. AR05-mCherry STm were then treated with the indicated antibodies at room temperature. Ten μL of cells was spotted on uncharged microscope slides at 10 min intervals for 30 min. Four to 7 images were taken for each condition at each time point at 20× magnification in both the DIC and Texas Red (600 ms of exposure/frame) channels on a Nikon TI inverted microscope equipped with a CoolSnap HQ2 camera (Photometrics, Tucson, AZ). Mean fluorescence intensity (MFI) per aggregate (MFI = mean gray value – (background mean gray value × area)) was quantified using Fiji (ImageJ 1.52p).^68,69^ To outline aggregates, the Texas Red channel was thresholded (minimum value of 157). Using this thresholded image, the mean gray value and area of the individual aggregates were measured on the original Texas Red channel. MFIs for all aggregates were averaged for each biological replicate.

### Animal Care and Ethics Statement

The mouse experiments in this study were reviewed and approved by the Wadsworth Center’s Institutional Animal Care and Use Committee (IACUC) under protocol #17-428. The Wadsworth Center complies with the Public Health Service Policy on the Humane Care and Use of Laboratory Animals and was issued assurance number A3183-01. The Wadsworth Center is fully accredited by the Association for Assessment and Accreditation of Laboratory Animal Care (AAALAC). Obtaining this voluntary accreditation status reflects that Wadsworth Center’s Animal Care and Use Program meets all standards required by law and goes beyond the standards as it strives to achieve excellence in animal care and use. Mice were euthanized by carbon dioxide asphyxiation followed by cervical dislocation, as recommended by the Office of Laboratory Animal Welfare (OLAW), National Institutes of Health.

### Mice

Female BALB/c mice age 8–12 weeks were purchased from Taconic Biosciences (Rensselaer, New York) and cared for by the Wadsworth Center Animal Core Facility. All experiments were performed in strict ordinance of the approved Wadsworth Center’s IACUC protocols as described above.

### STm Oral Challenge

Overnight cultures of AR04 and AR05 were inoculated as described and subcultured 1:50 in LB broth and adjusted to an OD_600_ of 0.7. AR04 and AR05 cultures were mixed 1:1 and washed twice in sterile PBS by pelleting cells at 6000*g* for 3 min. Challenge inoculum was resuspended in PBS and placed on ice prior to gavage. Bacterial input was plated on LB agar containing kanamycin (50 μg/mL) and X-gal (5-bromo-4-chloro-3-indolyl-β-d-galactopyranoside) (40 μg/mL) at the start of the experiment to determine ratios of AR04 and AR05. BALB/c female mice were orally administered 200 μL of antibody treatment in PBS immediately before a 200 μL dose of AR04/AR05 STm (4 × 10^7^ CFUs per mouse) via a 1 mL syringe and feeding needle (#01-208-87, Fisher Scientific) as already described.^36^ Animals were euthanized 24 h postinfection, and laparotomies were performed to isolate Peyer’s patches from the small intestine. Approximately 7–10 Peyer’s patches were collected from each mouse using curved scissors (#14061-09, Fine Science Tools, Foster City, CA) and pooled in 1 mL ice-cold sterile PBS. Tissue samples were homogenized in 2 mL tubes containing 2.8 mm zirconium ceramic oxide beads using a BeadMill 4 homogenizer (Fisher Scientific) three times for 30 s each at 3 m/s. Samples were placed on ice to cool between each round of homogenization. Homogenates were serially diluted and plated (100 μL/plate) on LB agar containing kanamycin (50 μg/mL) and X-gal (40 μg/mL) and were incubated overnight at 37 °C. Blue and white colonies were counted to compute competitive indices (CIs) of AR04 and AR05 isolated from each mouse (CI = [(% strain A recovered/% strain B recovered)/(% strain A inoculated/% strain B inoculated)]). All samples that contained less than 30 CFUs (per 100 μL) were considered “too few to count” (TFC) and were not included in the data set.

For analysis by histology and immunohistochemistry, overnight cultures of ATCC14028 were prepared as described above. Cohorts of BALB/c female mice were orally administered 200 μL of PBS, Sal4 IgG, or Sal4 SIgA at the indicated concentrations before an oral challenge of ATCC14028 (∼4 × 10^7^ CFUs). Groups of animals in each treatment group were then euthanized either 20 or 40 min postinfection. A laparotomy was performed on each animal, and the entire gastrointestinal tract was quickly removed, placed in histology cassettes, and fixed in buffered formalin for 24 h. After 24 h, cassettes were transferred to 70% ethanol solution until paraffin-embedding

### Staining of STm–mAb Complexes in Bovine Thrombin Clots

ATCC14028 overnight cultures were prepared as described above. Cells were treated with PBS, Sal4 IgG, or SIgA at the indicated concentrations and pelleted at 6000*g* for 3 min. Antibody-treated pellets were resuspended in bovine plasma (Sigma-Aldrich) and dispensed into cryomolds precoated with bovine thrombin (Sigma-Aldrich) on ice to initiate clotting, similar to that already described.^45^ Plasma clots were incubated at 4 °C for 20 min, removed from molds, and wrapped in lens paper prior to placement in histology cassettes. Cassettes were fixed in buffered formalin and transferred to 70% ethanol prior to paraffin-embedding and sectioning, as described above. Slides were counterstained using hematoxylin and eosin (H&E).

### Immunohistochemistry of STm-Inoculated Mouse Tissues

Paraffin-embedded tissue samples were sectioned at a thickness of 3–4 μm on charged microscope slides. Samples were deparaffinized using CitriSolv (Decon Laboratories, Inc., King of Prussia, PA) and rehydrated sequentially in graded alcohols. Antigen retrieval was performed by incubating slides in 10 μg/mL proteinase K (MilliporeSigma) in PK buffer (0.6 M Tris (pH 7.5)/0.1% CaCl_2_) for 10 min at RT. Blocking of endogenous peroxidase and alkaline phosphatase was performed by incubating slides in Rodent Block M (BioCare Medical, Pacheco, CA) followed by incubation with BLOXALL Endogenous Blocking Solution (Vector Laboratories, Burlingame, CA). Slides were washed in TBS buffer and incubated with primary rabbit Salmonella O Antiserum (Group B Factors 1, 4, 5, 12, #BD 229481, Becton, Dickinson and Company) for 1 h at 1:5000 dilution. Slides were then incubated in AP-polymer (Rabbit on Rodent AP-Polymer, BioCare Medical) followed by Vina Green Chromogen (BioCare Medical, Pacheco, CA) treatment. Tissues were finally counterstained with hematoxylin (BioCare Medical) before mounting with EcoMount (BioCare Medical). Slides were cured at 60–70 °C for 15 min.

### Crystal Violet Assay

Crystal violet (CV) assays were done as previously described.^70^ Indicated strains of *Salmonella* Typhimurium cells were grown to mid log phase (OD_600_ ∼ 0.6) and diluted 1:2 into LB medium containing indicated antibodies at 50 μg/mL for 1 h at 37 °C in shaking conditions (220 rpm) in borosilicate glass tubes with cork stoppers. Tubes were subsequently washed three times with PBS and then fixed with methanol for 15 min. Once dried, tubes were stained with 0.1% crystal violet dye for 5 min and then rinsed with water. CV was solubilized with 30% acetic acid for 30 min. To quantitate CV staining, 200 μL of solubilized CV was transferred to a microtiter dish and the *A*_550_ was read using a Versamax Microplate Reader. Images were taken after incubation with antibody before the first wash step and post-CV staining.

### Statistical Analysis and Graphics

For statistical assessment, the indicated methods of analysis were performed using GraphPad Prism 8 software (San Diego, CA). Graphical diagrams were designed using BioRender.com.

## Supporting Information Available

Characterization of human recombinant Sal4 mIgA and dIgA mAbs; Sal4 mAbs specific for the O5-polysaccharide; Sal4 mIgA and dIgA agglutinate live STm cells by flow cytometry; Sal4 SIgA blocking STm entry into Peyer’s patches at time of challenge; Sal4 SIgA-treated STm cells, refractory to detection by immunohistochemistry *in vivo* (PDF)
