# Supplementary material for: Recombinant Human Secretory IgA Induces Salmonella Typhimurium Agglutination and Limits Bacterial Invasion into Gut-Associated Lymphoid Tissues
Source: ACS Infect Dis. 2021 Mar 17;7(5):1221–35. doi: 10.1021/acsinfecdis.0c00842 (PMC8154420; doi:10.1021/acsinfecdis.0c00842)
Supplement: Supplementary file 1 — id0c00842_si_001.pdf [file id0c00842_si_001.pdf]

Supporting Information

Supplemental Figures S1-S5, pages S1-S6

Recombinant Human Secretory IgA Induces *Salmonella* Typhimurium Agglutination and Limits Bacterial Invasion into Gut-Associated Lymphoid Tissues

Angelene F. Richards[1, 2], Danielle E. Baranova[2], Matteo S. Pizzuto[3], Stefano Jaconi[3], Graham G. Willsey[2], Fernando J. Torres-Velez[2], Jennifer E. Doering[2], Fabio Benigni[3], Davide Corti[3], and Nicholas J. Mantis[1, 2*]

[1]Department of Biomedical Sciences, University at Albany School of Public Health, Albany, NY 12208; [2]Division of Infectious Diseases, Wadsworth Center, New York State Department of Health, Albany, NY 12208; [3], [4]Humabs BioMed SA a Subsidiary of Vir Biotechnology Inc., 6500 Bellinzona, Switzerland

*Corresponding author:

Nicholas J. Mantis, Ph.D.

Phone: (518) 473-7487 email: nicholas.mantis@health.ny.gov

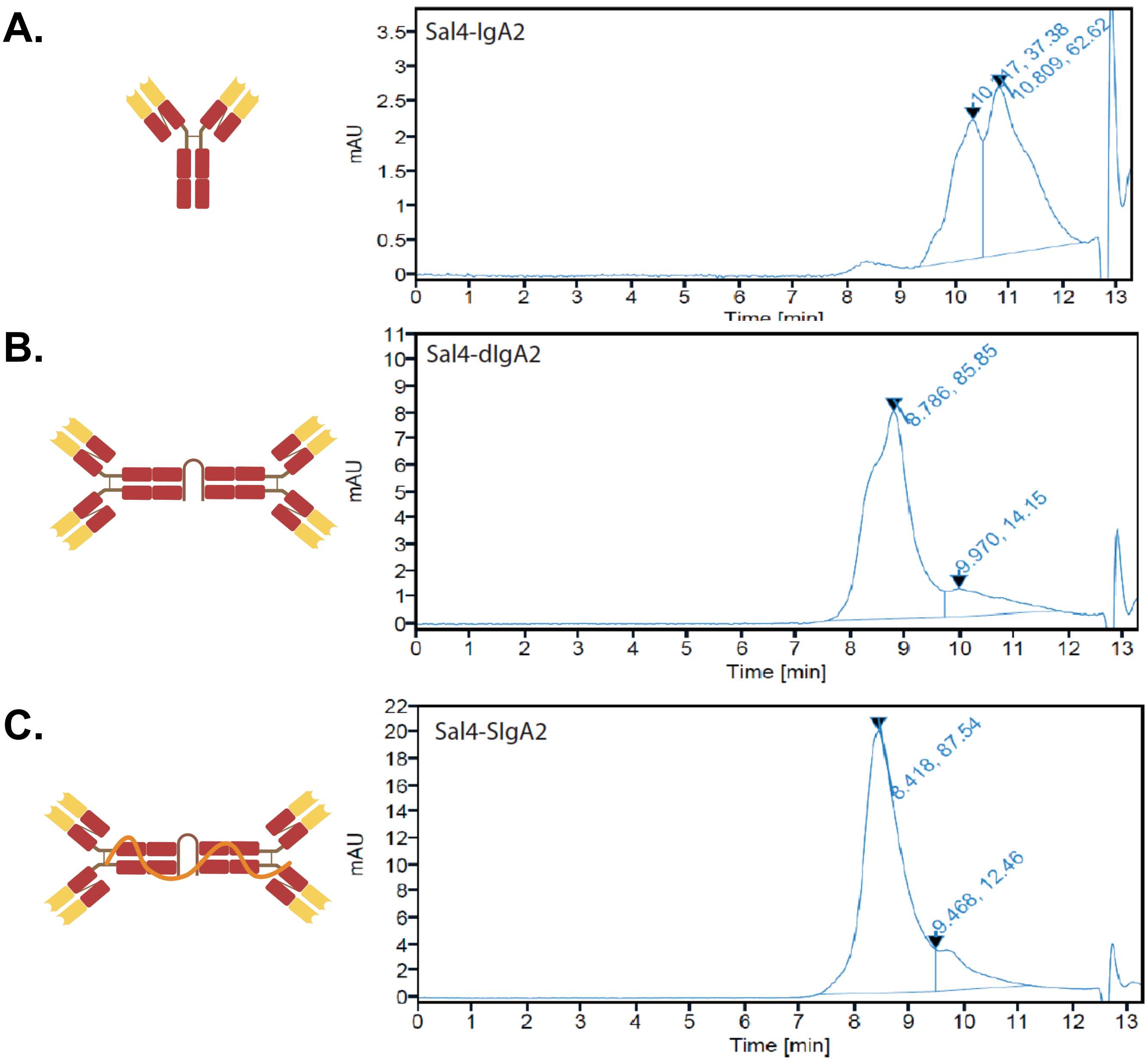

Figure S1. Characterization of human recombinant Sal4 mIgA and dIgA mAbs. (A) SEC-UHPLC of purified (A) Sal4 mIgA, (B) dIgA , and (C) SIgA, confirming correct isoform size.

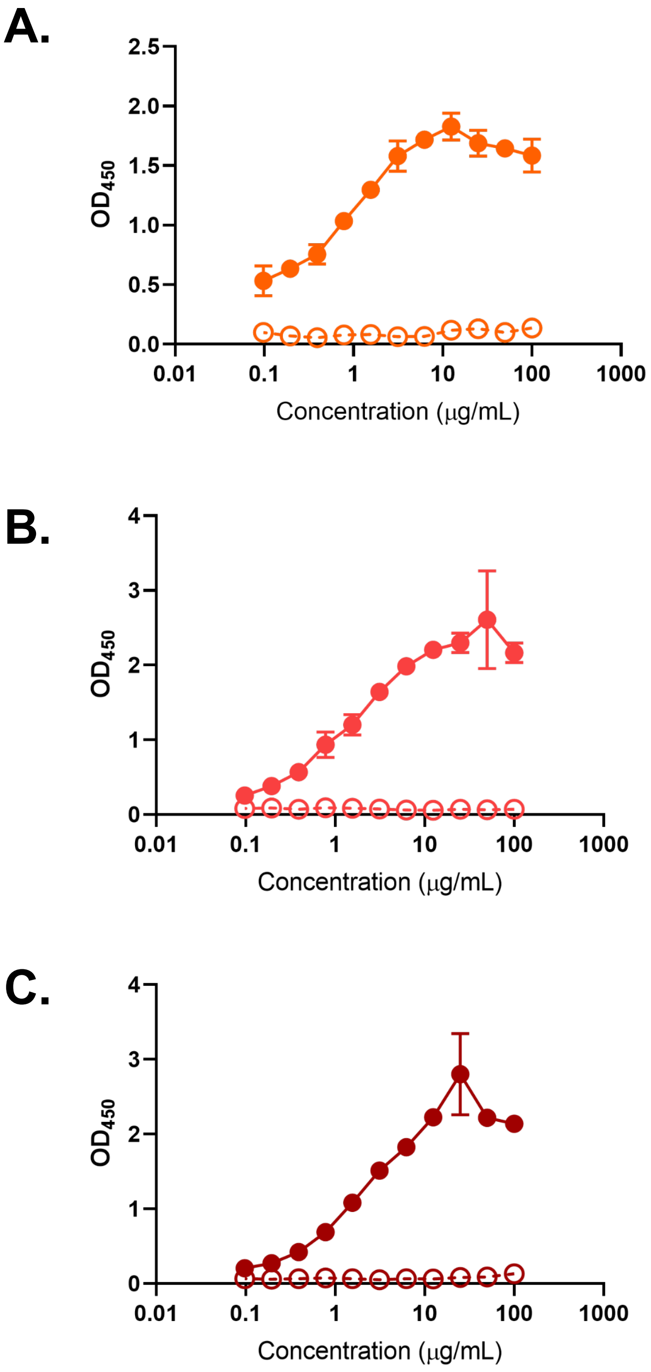

Figure S2. Sal4 mAbs are specific for the O5-polysaccharide. Sal4 mAbs (A) Sal4 mIgA, (B) dIgA, and (C) SIgA, react with STm wildtype strain AR05 (filled circles) but not mutant strain AR04 (open circles), which lacks the O5-epitope, by whole-cell ELISA. Graphs depict two technical replicates and are representative of two biological replicates.

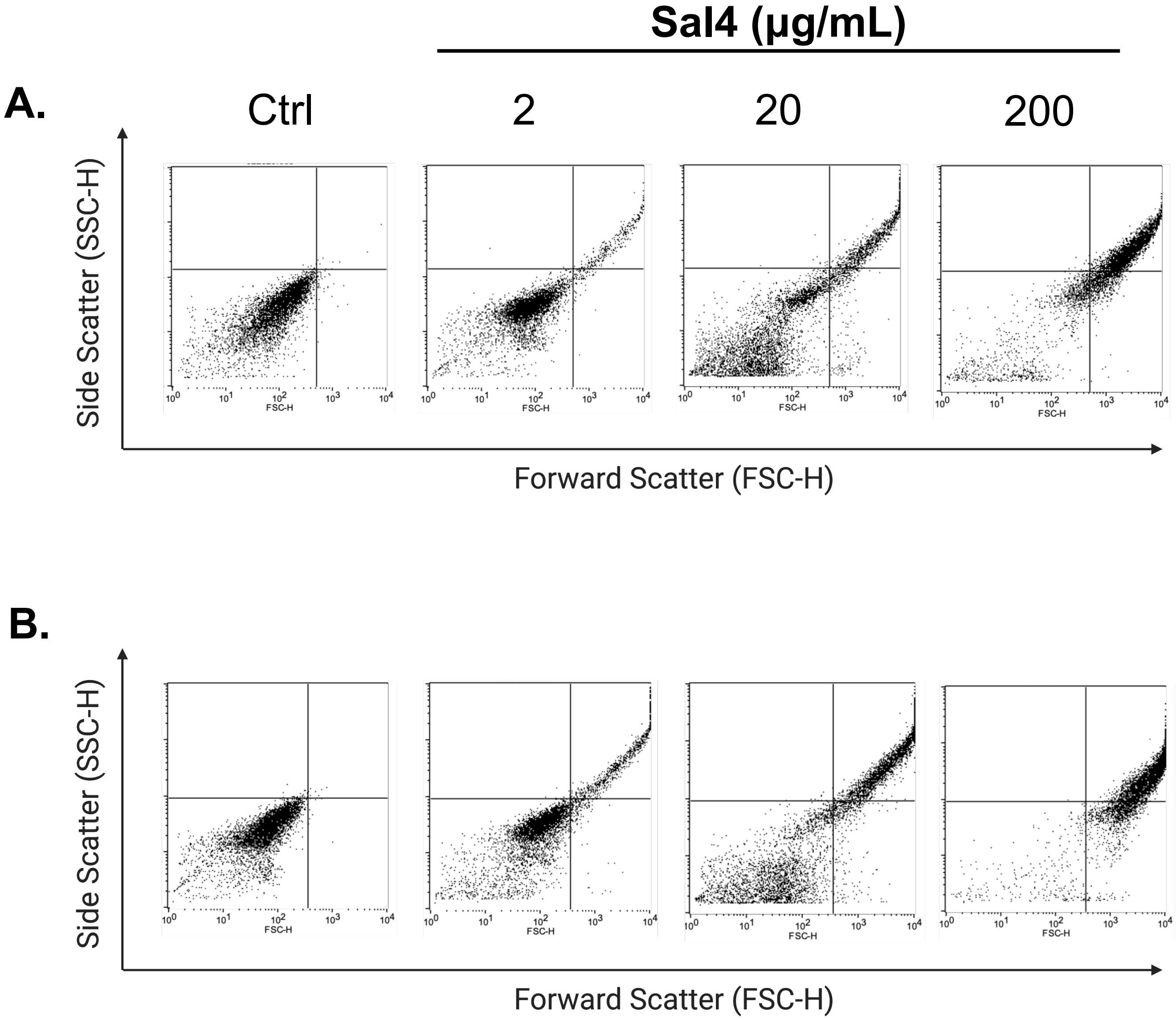

Figure S3. Sal4 mIgA and dIgA agglutinate live STm cells by flow cytometry. Mid-log phase cultures of AR05 were washed in PBS and incubated with 2, 20, or 200 μg/mL Sal4 mIgA, dIgA, or control SIgA for 1 h at 37°C. 10,000 events per sample were analyzed on a BD FACSCalibur (BD Biosciences, San Jose, CA) by forward scatter (FSC) and side scatter (SSC) to visualize aggregate size and granularity, as described previously. Representative flow cytometry plots showing (A) Sal4 mIgA and (B) Sal4 dIgA. Results show representative plots from three separate biological experiments.

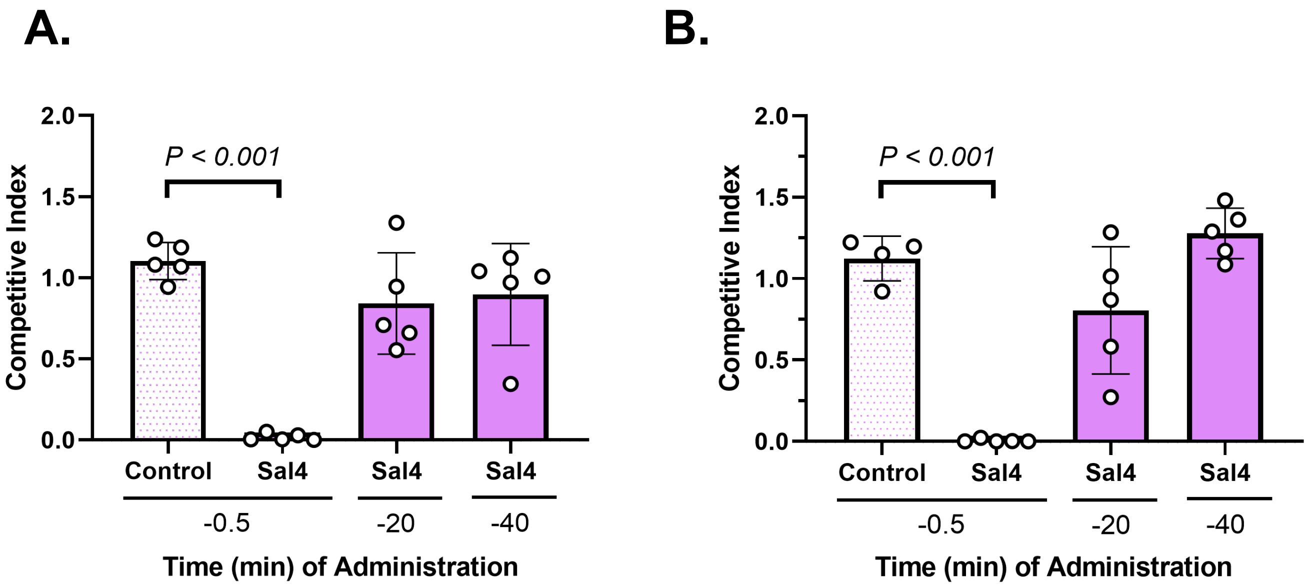

Figure S4. Sal4 SIgA blocks STm entry into Peyer's patches at time of challenge. (A & B). BALB/c female mice were orally administered 50 µg Sal4 SIgA or isotype control antibody in (A) PBS or (B) sodium bicarbonate and protease inhibitors either immediately before, 20 or 40 min before a 1:1 challenge with AR04 and AR05 STm strains (~4 x 10^7 CFUs). 24 h post-infection Peyer's patches were isolated, homogenized, and plated, as described. Shown are the results of two separate experiments with at least 4 mice per group. Statistical significance was assessed by one-way ANOVA followed by Tukey's post-hoc multiple comparisons test.

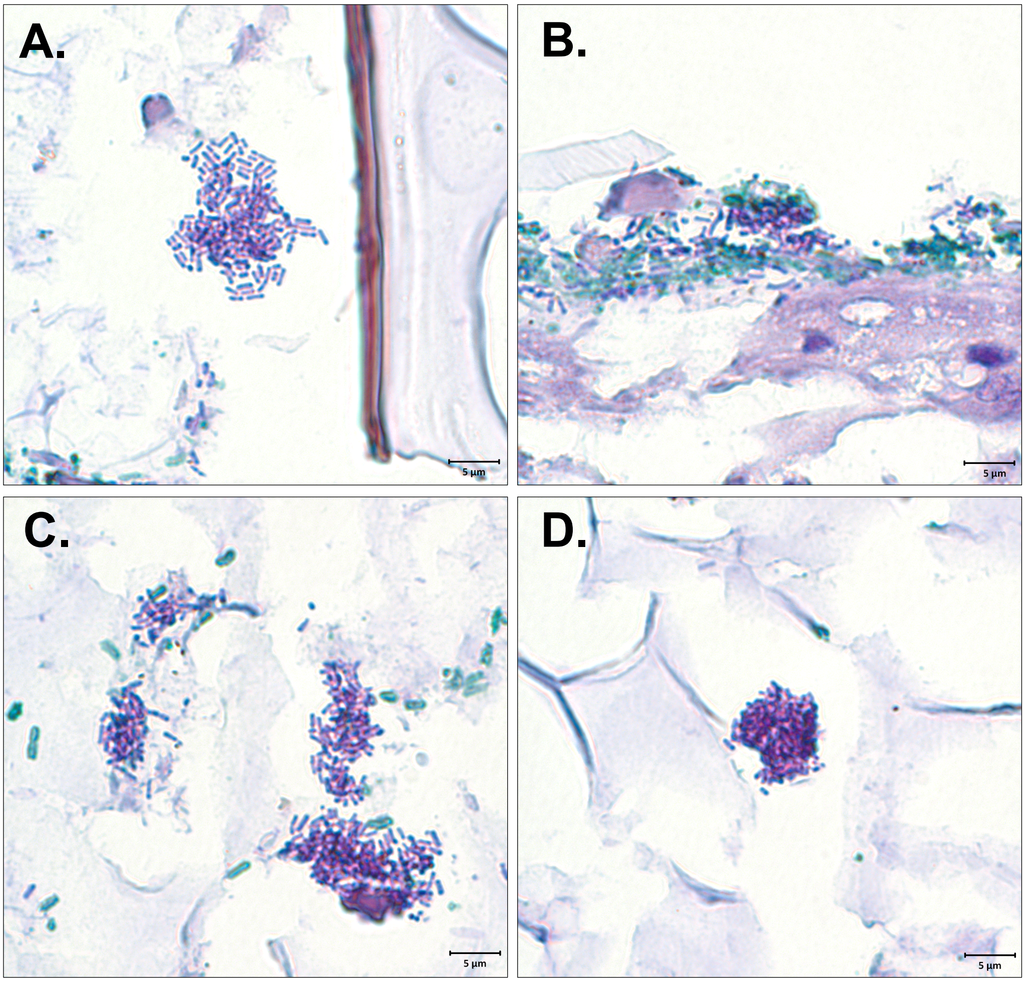

Figure S5. Sal4 SIgA-treated STm cells are refractory to detection by immunohistochemistry *in vivo*. (A-D) Sal4 SIgA treatment induced robust agglutination in the intestines of mice challenged with STm strain ATCC14028 40 min post-infection and was associated with reduced staining of STm cells by rabbit Salmonella Group B-specific antiserum (BD Difco). Positively stained cells (green) were primarily found at the periphery of STm aggregates within the lumen. Cells at the center of aggregates were resistant to staining and chromogen detection. Scale bars indicate 5 µm.
